# Development of a Multispectral Image Database in Visible–Near–Infrared for Demosaicking and Machine Learning Applications

**DOI:** 10.3390/jimaging12010002

**Published:** 2025-12-20

**Authors:** Vahid Mohammadi, Sovi Guillaume Sodjinou, Pierre Gouton

**Affiliations:** ImViA Laboratory, UFR Sciences et Techniques, Université Bourgogne Europe, 21000 Dijon, France

**Keywords:** multi-spectral filter array, demosaicking, annotated image database, segmentation, deep learning

## Abstract

The use of Multispectral (MS) imaging is growing fast across many research fields. However, one of the obstacles researchers face is the limited availability of multispectral image databases. This arises from two factors: multispectral cameras are a relatively recent technology, and they are not widely available. Hence, the development of an image database is crucial for research on multispectral images. This study takes advantage of two high-end MS cameras in visible and near-infrared based on filter array technology developed in the PImRob platform, the University of Burgundy, to provide a freely accessible database. The database includes high-resolution MS images taken from different plants and weeds, along with annotated images and masks. The original raw images and the demosaicked images have been provided. The database has been developed for research on demosaicking techniques, segmentation algorithms, or deep learning for crop/weed discrimination.

## 1. Introduction

Multispectral (MS) imaging provides rich spectral information through high-resolution spatial images, using either single-shot or multiple-shot acquisition methods. This capability makes it highly valuable in various domains such as medical diagnostics, food quality control assessment, object detection, and remote sensing. In recent years, MS imaging has received considerable attention in agriculture, especially in precision farming, where it supports tasks such as crop monitoring, weed detection, and stress analysis [1,2,3].

Traditionally, an MS image is represented as a cube of 3 to 8 spectral bands, typically covering the visible (VIS) spectrum or extending into the ultraviolet (UV) or near-infrared (NIR) regions. The number of bands and the spectral regions selected depend largely on the target application and the required processing algorithms. The wide range of MS imaging applications includes information extraction, segmentation, object detection, classification, geometric measurements, and image fusion [4,5,6,7,8,9,10].

MS imaging has been growing rapidly and has responded to the needs of diverse fields [11,12,13,14,15,16,17]. There exist several types of MS image acquisition systems, such as Single Sensor with Filter Wheel, Array of Monochromatic Cameras, and Beam Splitter with Multiple Sensors. A recent advancement in MS imaging is the Multispectral Filter Array (MSFA) technology, which is an extension of the color filter pattern used in color cameras to include more spectral bands. Accordingly, MSFA-based cameras acquire a raw MS image in a single shot, where each pixel records a specific band. These images require dedicated demosaicking algorithms to reconstruct the full spectral cube [18]. This technology offers compact and inexpensive MS cameras, which makes it promising for many applications. Also, MS imaging based on MSFA technology reduces the mechanical complexity of the imaging system compared to set-ups needing separate filters or multiple cameras.

The significance of MS imaging for crop/weed segmentation and deep learning-based plant detection arises from the fact that the spectral response of each object is unique. Hence, by analysis of the images at specific spectral bands, it is more effective to detect and discriminate different plants. Borregaard et al. [19] employed line imaging spectroscopy from 670 to 1070 nm to separate potatoes, sugar beet, and several weed species; analysis of selected bands showed high recognition rates, with potato vs. weeds and sugar beet vs. weeds correctly classified in 94% and 87% of cases, respectively. Liu et al. [20] collected hyperspectral images from 380 to 760 nm over carrot seedlings and several common weeds; by working entirely in the VIS domain, they still achieved up to about 91% accuracy when all weeds were aggregated into a single class, suggesting that visible-only imaging can suffice where color and leaf structure differ strongly. Zu et al. [21] measured canopy reflectance of two cabbage cultivars and five grass and broadleaf weeds between 350 and 2500 nm, but band-selection analysis indicated that a carefully chosen subset of bands, heavily concentrated in the VIS and VNIR region, was enough to reach 100% classification accuracy, so full SWIR coverage was not essential for crop–weed separation. More recently, UAV-based datasets such as WeedsGalore have used discrete multispectral bands, including RGB, a red-edge band around 730 nm, and an NIR band around 840 nm, and transformer–CNN segmentation models using these bands have achieved mean IoU values near 79%, markedly outperforming RGB-only baselines and confirming the benefit of adding NIR and red-edge channels in the 700–900 nm region for robust crop–weed segmentation under variable field conditions.

The increasing demand for robust algorithms in these domains highlights the need for publicly available, high-quality MS datasets. However, such datasets remain scarce, particularly for MSFA-based systems, thus limiting progress in research such as demosaicking, segmentation, and deep learning applications. Hordley et al. [22] developed a small database of MS images, which was used for the comparison of an MS acquisition system based on interferometry and a spectral tele-spectroradiometer. The database included 22 images. Chang et al. [23] provided an MS image database for face recognition. The whole dataset included 2624 MS images. The MS acquisition system used consisted of a monochrome camera coupled with an electronically tuned LCTF (Liquid Crystal Tunable Filter). Lapray et al. [24] developed a polarimetric and multispectral image database that covered the VIS and NIR regions of the spectrum. Their acquisition system, based on a dual-RGB method, produced six spectral/polarimetric channels in each acquisition. The database includes a variety of objects such as plastic, painting, liquid, fabric, etc. In a recent study, Gutierrez-Navarro et al. [25] introduced an open-access MS image database for hand perfusion evaluation based on MSFA technology. The MS camera utilized in this study was a snapshot camera which could record 8 spectral bands in VIS and NIR. The database includes the MS images of the hands of 45 healthy participants.

This work introduces a novel MS image database designed to address the lack of MS image database based on MSFA technology. The dataset includes raw mosaicked images and corresponding annotations, enabling both unsupervised and supervised learning approaches. It comprises acquisitions from two MS cameras operating in the VIS and NIR ranges, capturing the same agricultural scenes. The proposed dataset is especially relevant for agricultural imaging applications, such as the following:

- Development and benchmarking of demosaicking and mosaicking techniques adapted to MSFA.
- Semantic segmentation of vegetation (e.g., crop vs. weed).
- Plant species classification and phenotyping under field conditions.
- Design and evaluation of deep learning models for multispectral agricultural tasks.

To the best of our knowledge, this is the first publicly available, free MS image dataset based on MSFA technology, specifically tailored for both general-purpose and agricultural image processing applications.

## 2. Materials and Methods

This section presents the details of the image acquisition system, the structure of the images, and the organization of the database. It aims to help researchers prepare their desired data from raw images and develop their own algorithms based on the dataset and the provided labeled images. Establishing a database of MS images involves several steps, including the design and development of MSFAs, development of the MS camera, creation of supporting hardware and software, image acquisition, and preliminary processing. These steps will be explained in the following sections.

### 2.1. Multispectral Acquisition Set-Up

**Figure 1:**
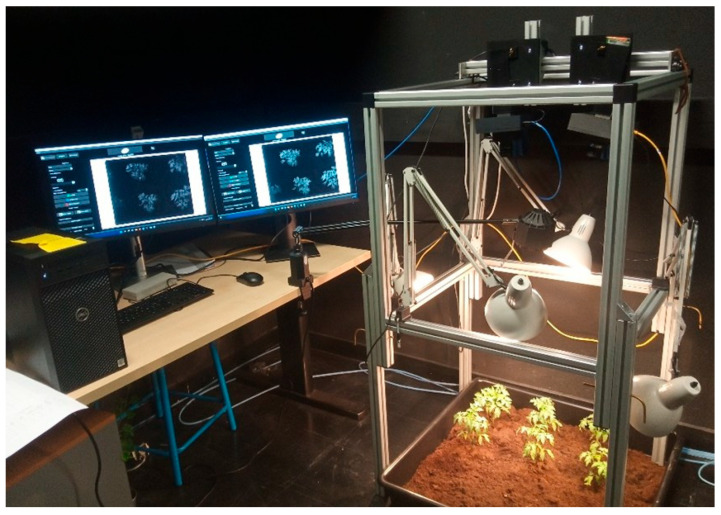
Set-up for MS image acquisition (this camera was supported by the EU PENTA/CAVIAR).

The development of the cameras was performed in the PImRob platform of ImViA (Image et Vision Artificielle) laboratory of the Université de Bourgogne Europe. The acquisition system consisted of two MS cameras covering VIS and NIR (Figure 1). The cameras capture 16 spectral bands (i.e., 8 bands for each) from 380 to 720 nm and from 680 to 950 nm for VIS and NIR, respectively. The acquisition set-up consisted of the cameras placed beside each other and three halogen lamps placed around the cameras. The cameras were controlled simultaneously by two software interfaces. The images have been saved in TIF format with a resolution of 1280 × 1024.

**Figure 2:**
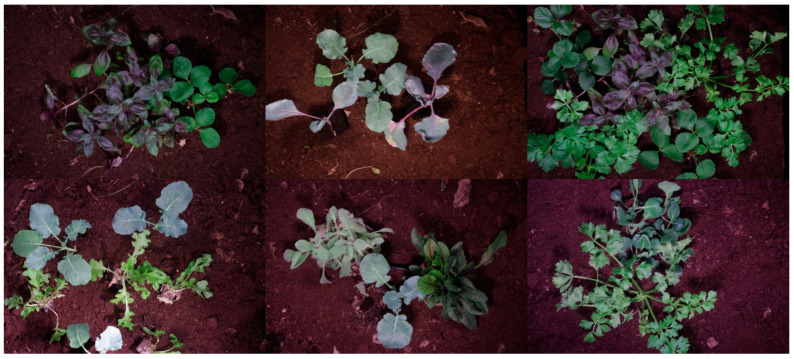
Color examples representing the scene where MS images were obtained.

The database includes MS images of four plants (i.e., tomato, cucumber, garden bean, and bell pepper) and five weed species (i.e., bindweed, edible sedge, Plantago lanceolata, potentilla, and sorrel). The plants were freshly imported to the lab for image acquisition. The plants were displaced by their root to the imaging scene. This could keep them healthy during image acquisition. Figure 2 represents several color examples of the scene. The database includes different arrangements of plants with sparse or dense imaging scenes.

### 2.2. Multispectral Filter Array (MSFA)

**Figure 3:**
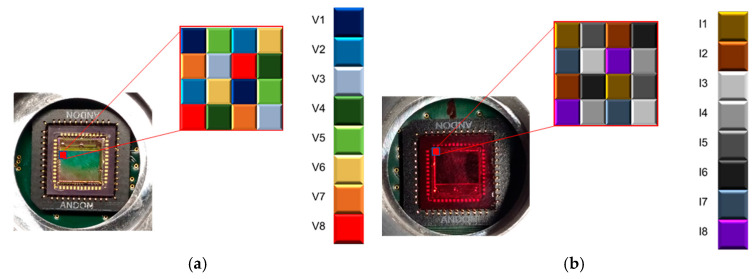
The architectures of MSFAs: (**a**) visible camera and (**b**) near-infrared camera.

MSFAs consisted of mosaics of filters arranged in a matrix where each filter is associated with a specific spectrum. In this work, a set of 8 filters based on the Fabry–Perot principle was used to cover the VIS and NIR regions [26]. The architectures of the MSFAs are presented in Figure 3. This figure shows the architecture for a single mosaic pattern, and the architecture has been repeated all over the image sensor. The architecture of the MSFA affects the spectral image reconstruction as it determines how many pixels will be associated with each band and how the missing information for each spectral band needs to be interpolated or estimated.

Table 1 provides the spectral details of the spectral bands for both VIS and NIR cameras. After demosaicking, the extracted images can be associated with their spectral bands for further processing. This information can be beneficial for the research for the detection and analysis of plants based on specific spectral bands. For example, in the VIS camera, band 7 has the center frequency of 646 nm, which is ideal for plants’ Chlorophyll b analysis (i.e., the peak absorption band is 642 nm). The table also shows the FWHM (Full Width at Half Maximum) that varies for different bands. Narrow FWHM leads to the capturing of light from a small wavelength range, which allows for more precise measurements. A wider FWHM represents that more information about different light wavelengths is present in the MS image. It is noted that the last band in the VIS camera and the first band in the NIR camera possess the same spectral center frequency.

Previous research represents that the selected bands can be used for effective discrimination of crops and weeds. Brown et al. [27] used 400–900 nm leaf reflectance in no-till corn and identified key wavelengths around 440, 530, 650, and 730 nm to discriminate cotton from several weed species, showing that both Chlorophyll-related visible bands and the red edge are informative for weed sensing. Jurado-Exposito et al. [28] exploited a 750–950 nm window to distinguish broadleaf weeds, sunflower and wheat seedlings, highlighting the power of NIR reflectance and early red-edge behavior for separating cereal crops from broadleaf weeds. Mao et al. [29] focused on 700–1100 nm for wheat and broadleaf weeds such as goosefoots and shepherd’s purse, reporting about 97% correct classification when using selected NIR bands, again confirming the diagnostic value of NIR for crop–weed differentiation at the seedling stage.

### 2.3. Hybrid Sensor

The choice of the CMOS sensor is of high importance as its performance directly impacts the quality of the final images. E2V (Teledyne Company, Saint-Egrève, France) was chosen as it had a minimum pixel size of 4.5 µm and an extended sensitivity to the near-infrared. This sensor has a frame rate of 50 fps and a bit depth of 10 bits. Next, the MSFAs were placed on top of the image sensors to develop the MS image sensors. And finally, the MS image sensors were assembled with other components such as electronic boards, cables, and camera cases.

### 2.4. Demosaicking

The image taken by the MS camera is a single-layer image providing reflective information in different spectral bands. For the reconstruction of images of each spectral band, it is necessary to extract the spectral information of each band and find the missing values. MS image demosaicking is a method that estimates missing pixels in different spectral channels of the mosaic image. This was performed using the bilinear demosaicking technique in MATLAB software (R2018b, the US). The reconstructed images are the same size as the original images.

To evaluate the computational performance of the proposed demosaicking method, we measured the average execution time on a set of three test images. Each image was processed using MATLAB on a PC equipped with a 13th Gen Intel^®^ Core™ i7-1370P processor running at 1.90 GHz and 32 GB of RAM. The average execution time observed was 0.85 s. The maximum memory usage during processing was approximately 125 MB.

### 2.5. Image Labeling

Preparing labeled images is a challenging and time-consuming task. There exist several techniques and tools to perform image labeling, including automatic or manual toolboxes as well as online platforms. In this study, the MATLAB Image Labeler application was used to manually label each image to achieve the highest possible precision. The application provides a user-friendly interface and robust features for accurate image annotation. To perform image labeling, the images are loaded into the application and begin by defining regions of interest (ROIs) around the objects of interest in each image. Labels are then assigned to each ROI to identify the corresponding objects, using the built-in text and selection tools. The manual drawing functionality also allowed us to draw contours around objects in the images, providing additional flexibility for annotation. Once the ROIs are defined and labels are assigned, it can organize the annotations into logical groups and export the labeled data in various formats compatible with the needs of our research or application project.

## 3. Discussion

### 3.1. Structure of the Database

The database has been organized in a way that facilitates the automatic reading of images. MS image, demosaicked images, binary masks, and the annotated image are in separate folders and all have been placed in a folder under image number. Then, all image folders are in the main database folder. The raw MS images have a resolution of 1280 × 1024 in TIF extension and saved in 8-bit format. The database provides a total of 512 images.

### 3.2. Demosaicking

**Figure 4:**
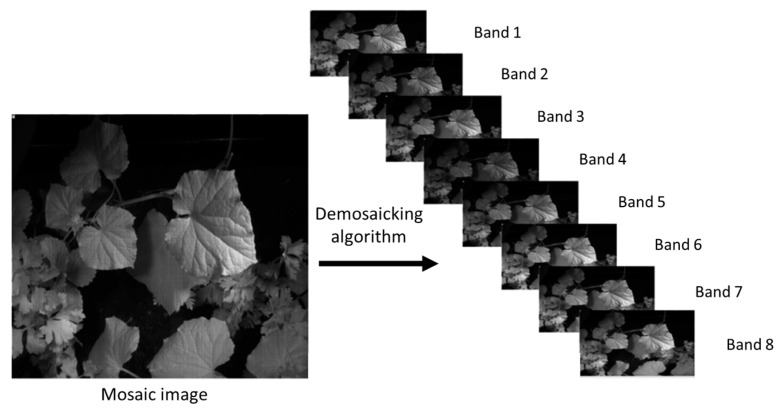
Raw image and its diagonally demosaicked version.

Demosaicking represents the final and crucial step in the multispectral image acquisition process, aiming to reconstruct the missing pixel values in a way that they are closest to the real value of those pixels. To reconstruct a full multispectral data cube from the raw mosaic produced by the Multispectral Filter Array (MSFA), we applied a band-wise bilinear demosaicking procedure adapted to the MSFA pattern (Figure 4). First, the MSFA layout was used to identify the spatial locations corresponding to each spectral band. For every band, a sparse 2D image was initialized by placing the measured pixel values at their corresponding positions and assigning zeros elsewhere. Each band image was then independently interpolated onto the full spatial grid using bilinear interpolation, where missing pixels were estimated as the weighted average of their four nearest neighbors in the horizontal and vertical directions. This approach leverages the local spatial smoothness commonly present in multispectral scenes while preserving the relative intensity consistency between neighboring pixels. Although bilinear interpolation is a simple and computationally efficient method, it provides a stable baseline reconstruction for MSFA-based imaging and enables subsequent evaluation against more advanced demosaicking techniques. The traditional demosaicking techniques include nearest-neighbor replication, bilinear interpolation, cubic spline interpolation, heuristic methods, wavelet-based methods, and so on [30,31,32]. Recently, deep learning methods have been gaining much attention for demosaicking [33,34,35,36,37,38,39,40,41]. However, simple interpolation extensions (bilinear, guided filtering, and pseudo-panchromatic (PPAN) strategies) remain attractive for their low complexity and reasonable spatial preservation when a dense reference band exists; recent generic PPAN-based algorithms show robust spectral consistency across varied MSFA layouts but can blur fine spatial details when spectral sampling is very sparse [42]. Model-based approaches (sparse coding, iterative linear solvers, and physics-informed reconstructions) explicitly enforce spectral priors and inter-band correlations, yielding higher spectral fidelity and better preservation of narrow absorption features at the cost of heavier computation and parameter tuning; iterative methods that estimate pseudo-panchromatic images or jointly solve for spatial and spectral components are particularly effective when training data are limited [43].

On the other hand, deep learning methods (2D/3D CNNs, residual networks, and attention modules) now dominate top performance in benchmarks: they recover fine spatial details and reduce aliasing/artifacts when large, representative training sets are available, but they are computationally expensive to train, can suffer spectral distortion if loss functions do not explicitly penalize spectral errors, and generalize poorly across sensor types unless domain adaptation or paired RGB/MS capture strategies are used [44]. Recent work therefore emphasizes hybrid designs—e.g., using a fast interpolation/PPAN initializer followed by a light-weight learned refinement or a sparsity/physics prior—to balance spectral fidelity, spatial resolution, and practical compute/memory constraints for real snapshot multispectral cameras [45].

Yu et al. [46] used a two-step method for demosaicking MS images based on MSFA technology. The first step was to apply a weighted bilinear interpolation convolution filter and then use the multi-scale dense connections large kernel attention method (MDLKA) to obtain high-resolution images. Liu et al. [47] proposed a deep learning method based on pseudo-panchromatic images for demosaicking of snapshot MS images. The proposed technique included two networks: Deep PPI Generation Network (DPG-Net) and Deep Demosaic Network (DDM-Net). They reported that the proposed method outperformed previous traditional and deep learning methods.

### 3.3. Annotated Images

**Figure 5:**
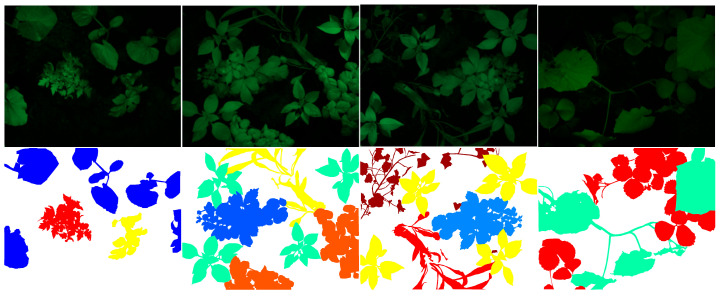
Examples of annotated ground truth images.

The database provides the annotated images for all MS images in the database. Figure 5 shows examples of the annotated images and the corresponding RGB images. In these images, all the spots of the same color belong to a specific plant. These labels can be utilized during the training and evaluation of deep networks. As the figure shows, the annotation has been performed precisely, especially on the edges of leaves and the critical curvatures. These annotated images are necessary for many segmentations and deep learning techniques for the training stage and testing the models. By incorporating these ground truth images into the training process, models can learn to accurately distinguish between different object classes, estimate their positions, and recognize the complex patterns.

**Figure 6:**
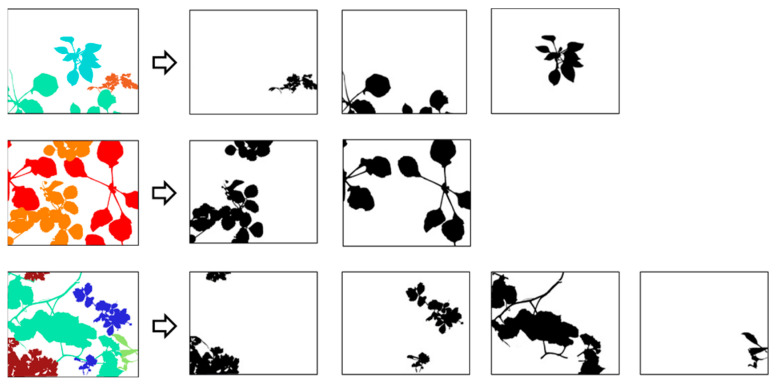
Examples of extraction of binary masks from labeled images.

In addition to the annotated images, we have provided binary masks in the database (Figure 6). These masks are images of the same size as the original images and provide labels corresponding only to a single plant. These masks will help researchers to build their dataset more easily for deep learning applications, as each plant can be introduced separately to the network.

It is worth noting that while the database has been prepared for demosaicking, segmentation, and deep learning research, it can serve specifically agricultural research for different purposes and applications, including plant detection, leaf counting, single-leaf segmentation, and so forth. Work remains to be performed on the extension of the database, increasing spectral bands, extension of the spectral range, improving the volume, the number of plants, etc.

### 3.4. Comparison with Other Datasets

To better position our contribution within the context of existing multispectral datasets, we compared our MSFA-based database with three widely used public datasets: the CAVE dataset, the Harvard dataset, and the NTIRE 2020 dataset. Table 2 presents this comparison in terms of the number of images, spatial resolution, number of spectral bands, spectral range, acquisition technology, and availability of annotations. Unlike CAVE and Harvard, which rely on rotary filter systems with sequential image acquisition, our database uses an MSFA approach, enabling single-shot acquisition through spatial mosaicking. This technology is particularly well suited for dynamic scenes and applications requiring high-speed image capture.

The database consists of 512 high-resolution mosaicked images (1280 × 1024 pixels), each comprising 16 spectral bands—8 in the visible range (400–720 nm) and 8 in the near-infrared (660–950 nm). The images are provided in TIFF format, along with pixel-level annotated ground truth to support supervised learning tasks. To our knowledge, this is the only publicly available MSFA-based dataset that combines high-resolution imaging, precise annotation, and a wide spectral coverage (400–1100 nm). In comparison,

- The CAVE dataset contains only 32 images, limited to the visible spectrum (400–700 nm).
- The Harvard dataset offers 50 images, with a slightly extended range (420–720 nm).
- The NTIRE 2020 dataset includes 480 images, but annotations are limited to specific subtasks, and the image resolutions vary.

This comparison highlights the added value of our dataset for agricultural research, plant segmentation, and the development of deep learning methods based on MSFA technology.

## 4. Conclusions

Due to the need for MS image databases, this work was undertaken to provide a free database dedicated to demosaicking and plant/weed discrimination based on deep learning applications. The database is based on MSFA technology as one of the most recent technologies in MS imaging, making it a valuable resource for research on MSFA design, optimization, and demosaicking algorithms. The database includes original MS images in visible and near-infrared, each composed of eight spectral bands. Both VIS and NIR images have the same MSFA pattern and can be perfectly utilized for demosaicking techniques. A diverse set of plants was used to construct scenes with significant variability. The MS images come with the annotated images to make them usable for both supervised and unsupervised segmentation or deep learning techniques. Future work will focus on expanding the diversity of plant species, integrating advanced calibration procedures, and offering ready-to-use benchmarks to promote reproducibility and facilitate collaboration within the research community.

## Figures and Tables

**Table 1:** Details of the spectral bands of MSFAs of both cameras.

Band	VIS Camera		Band	NIR Camera	
	Center Frequency (nm)	FWHM (nm)		Center Frequency (nm)	FWHM (nm)
1	429	71	1	680	31
2	465	52	2	712	30
3	498	44	3	739	30
4	529	39	4	772	29
5	571	36	5	812	30
6	606	34	6	847	28
7	646	33	7	872	27
8	680	33	8	905	27

**Table 2:** Comparison of MS Image Databases.

Database	Images	Resolution	Bands	Spectral Range (nm)	Spectral Step	Acquisition Method	Annotations	Public Access
CAVE	32	512 × 512	31	400–700	10 nm	Filter Wheel (Sequential)	No	Yes
Harvard	50	Varies	31	420–720	10 nm	Filter Wheel (Sequential)	No	Yes
NTIRE 2020	480	Varies	31	400–700	10 nm	Filter Wheel (Sequential)	Partial	Yes
Proposed database	512	1280 × 1024	16	400–1000	25–43 nm	MSFA	Yes	Yes

## Data Availability

The raw data supporting the conclusions of this article will be made available by the authors on request. The database will be downloadable via a direct download link under request: https://multispectraldatabase.vercel.app/ (accessed on 16 December 2025).
